# Storage and erasure of behavioural experiences at the single neuron level

**DOI:** 10.1038/s41598-019-51331-5

**Published:** 2019-10-14

**Authors:** T. L. Dyakonova, G. S. Sultanakhmetov, M. I. Mezheritskiy, D. A. Sakharov, V. E. Dyakonova

**Affiliations:** Koltzov Institute of Developmental Biology RAS, Vavilov St. 26, 119334 Moscow, Russia

**Keywords:** Cellular neuroscience, Neurophysiology, Animal physiology

## Abstract

Although predictions from the past about the future have been of major interest to current neuroscience, how past and present behavioral experience interacts at the level of a single neuron remains largely unknown. Using the pond snail *Lymnaea stagnalis* we found that recent experience of terrestrial locomotion (exercise) results in a long-term increase in the firing rate of serotonergic pedal (PeA) neurons. Isolation from the CNS preserved the “memory” about previous motor activity in the neurons even after the animals rested for two hours in deep water after the exercise. In contrast, in the CNS, no difference in the firing rate between the control and “exercise-rested” (ER) neurons was seen. ER snails, when placed again on a surface to exercise, nevertheless showed faster locomotor arousal. The difference in the firing rate between the control and ER isolated neurons disappeared when the neurons were placed in the microenvironment of their home ganglia. It is likely that an increased content of dopamine in the CNS masks an increased excitation of PeA neurons after rest: the dopamine receptor antagonist sulpiride produced sustained excitation in PeA neurons from ER snails but not in the control. Therefore, our data suggest the involvement of two mechanisms in the interplay of past and present experiences at the cellular level: intrinsic neuronal changes in the biophysical properties of the cell membrane and extrinsic modulatory environment of the ganglia.

## Introduction

Past experience, especially an unusual or stressful one, can be memorized by an organism and affect its “predictive models” of future events. This memory can impact the internal state and behavioral decisions for a long time. The fact, widely accepted by psychologists and human physiologists, receives notable support from studies in animal models^1–4^. Recently, it was demonstrated that even a comparatively simple invertebrate organism such as a mollusk uses memories of past experiences to inform decisions^5,6^.

Surprisingly, little is known about how past and present experiences interact at the level of a single neuron. The idea that the key mechanism of memory formation is based on altered synaptic weights in the neuronal circuit has inspired generations of experimental and theoretical work and prevails in understanding the mechanisms of brain plasticity in general. In recent years, several research papers challenged this view, arguing the existence of memory mechanisms at the level of an individual neuron^7–18^. However, in a mammalian neuronal network, it is often difficult to directly demonstrate the memory trace within a single neuron and elucidate its dependence on network influence. Possible involvement of all kinds of non-synaptic events, including extrasynaptic neuromodulatory influences in the mechanisms of past experience storage, has not been well elucidated.

The nervous system of mollusks provides a unique opportunity to directly investigate the interactions between a single cell and a neuronal network. Identified mollusk neurons can be isolated from the network to test whether circuit-level interactions or intrinsic cellular mechanisms underlie the phenomena observed at the system level^12,19^. Moreover, isolated neurons can be used as movable biosensors to monitor the extrasynaptic release of neuromodulators from certain parts of the nervous system^20^. This method helps elucidate whether synaptic or extrasynaptic mechanisms underlie a circuit-level interaction.

Earlier, in the mollusk *L. stagnalis*, we found that forced muscular locomotion (exercise) in low water produces long-term changes in the behavior and cell activity^21^. Previous exercise affected the behavioral state and decision-making of animals in a new environment and produced an excitatory effect on the activity of the serotonergic neurons controlling locomotion. Here, we used this simple model of the memory trace of previous exercise to clarify possible underlying mechanisms of experience storage at the cellular level. Contribution and interplay of mechanisms that are intrinsic and extrinsic to the pedal serotonergic neurons were the focus of our investigation.

We found that two hours of forced terrestrial locomotion (exercise) produced long-term changes in the electrical activity of pedal serotonergic neurons (PeA) that are preserved even after isolation of a neuron from the pedal ganglion. Two hours of “rest” in normal aquatic conditions following intense locomotion abolished the effects of exercise on serotonergic cell activity in the CNS but not after isolation of neurons from the CNS. To investigate whether the extrasynaptic microenvironment of pedal ganglia contributed to the above masking effect of CNS on the PeA neurons after rest we placed isolated control (non-exercised) and ER neurons close to their home ganglia. There were no significant differences in the activity between control and experimental isolated neurons in these conditions. When neurons isolated from control nervous systems were brought next to control and ER ganglia, a significantly stronger excitatory effect was detected in their response to the microenvironment of control ganglia than to the “rested after exercise” pedal ganglia. This finding supports our suggestion that rest after the exercise changes the pedal ganglia microenvironment content. Among other neurotransmitter ligands we tested were the effects of dopamine receptor antagonist sulpiride on PeA neurons. Sulpiride produced sustained excitation in PeA neurons from ER snails but not in the control group. Therefore, the increased content of dopamine in the CNS is likely to mask the excitatory state of PeA neurons after rest. We conclude that past experience can be stored within the neuron while the present context may control individual cell memory manifestation via changes in the neurochemical microenvironment of the neuron.

## Results

### Previous motor activity produces long-term excitation of serotonergic PeA neurons in the central ganglia and after complete isolation

**Figure 1:**
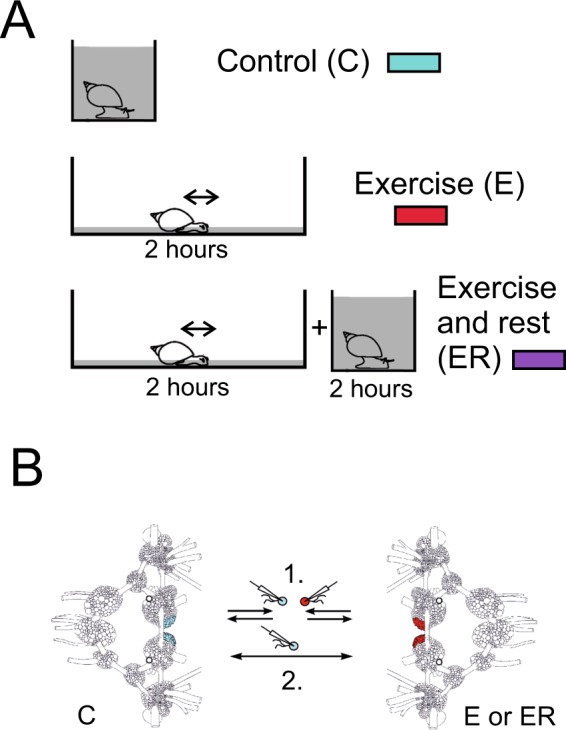
Schematic illustration of the experimental approach. (**A**) Procedure for the investigation of the effects of enhanced motor activity (exercise) and rest after exercise based on the method described in^21^. Snails were divided into two groups and resided individually for two hours in similar light conditions. Control group snails (C, marked with blue color) were kept in a cylinder filled with up to 9 cm of water to be able to use ciliary locomotion. Snails of the “exercise” experimental group (E, marked with red color) were kept in a 25 × 50 cm container containing a 1 mm layer of water which protected them from drying but forced them to use intense muscular locomotion. Snails of the “rest after exercise” experimental group (ER, marked with purple colour) were placed into in a cylinder filled with 9 cm of water to be able to use ciliary locomotion for 2 hours after 2 hours of exercise in low water. The procedure is modified after Korshunova, *et al*.^21^. (**B**) The procedure used for the investigation of modulatory effects of ganglia microenvironment based on method described in^12^. The neuron impaled with the microelectrode was isolated and moved away from the ganglion and placed in the middle between the control and experimental ganglia. Two approaches were used. (1) The neurons isolated from the control and experimental preparations were moved back close to their positions in their home pedal ganglia at a distance less than half-cell size (20–25 µm) and kept in this position for about two minutes; (2) Isolated neuron was first moved to the pedal cluster of experimental preparation, once again placed between the experimental and control ganglia, and then moved to the PeA cluster of the control preparation ganglia. The procedure was repeated several times.

**Figure 2:**
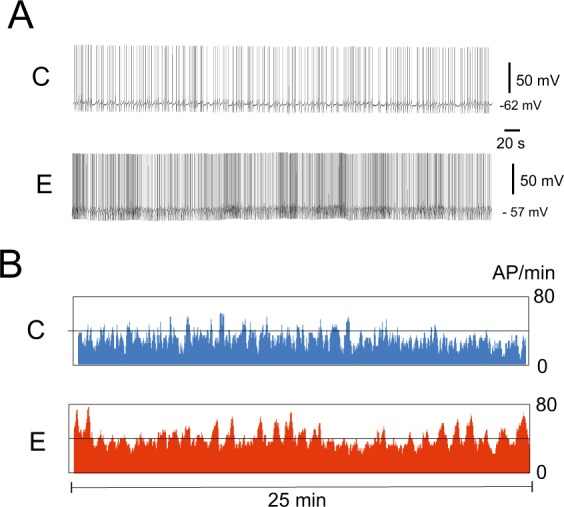
The effect of locomotion in low water (exercise) on the activity of PeA neurons in an isolated CNS. (**A**) Records of PeA8 neuron electrical activity in CNS from exercised (E) and control snails (C). The MP value is measured at the end of the records[procedure??]. (**B**) The frequency of action potentials per minute of PeA8 neurons for 25 minutes of recording from exercised (E) and control snails (C), first derivative without smoothing.

**Figure 3:**
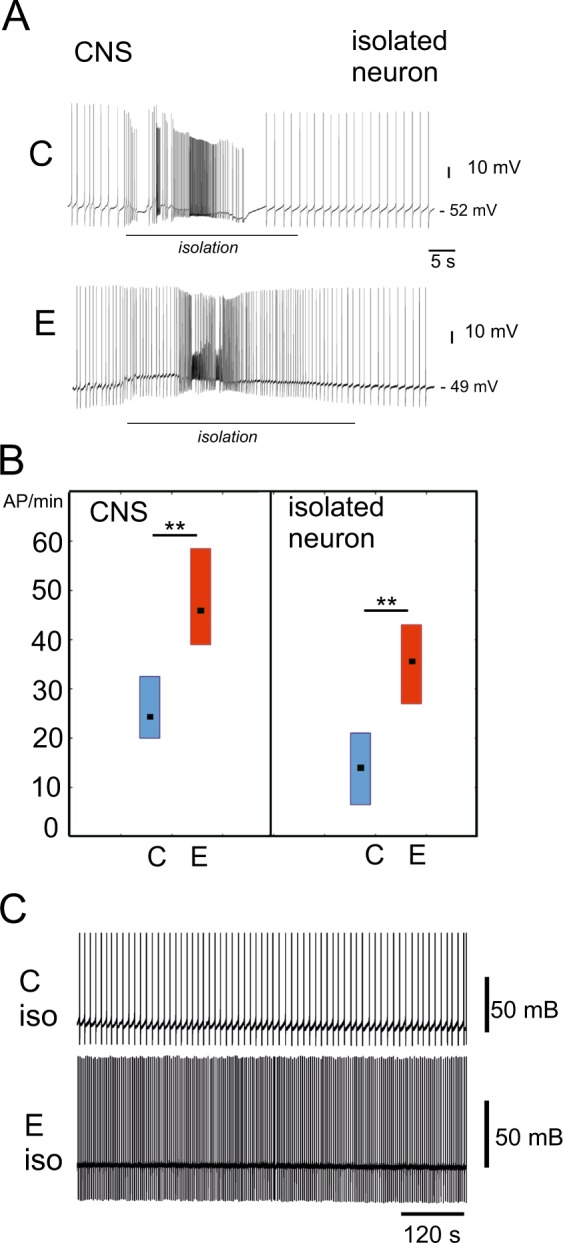
The effect of locomotion in low water (exercise) on the activity of PeA neurons in an isolated CNS and after complete isolation. (**A**) Records of control and exercised PeA neurons during isolation from the nervous system. The red line indicates mechanical isolation (electrode touch by the experimenter, pulling the neuron out of the ganglion, and moving the neuron away from the ganglion chemical microenvironment). The activity of control and exercised neurons in the CNS (left of the black line), and the activity of the same neurons in complete isolation (right of the black line) is shown. Isolation results in the decreased firing rate and hyperpolarized MP in both the control and the experimental cells. The MP volume is shown for isolated cells. Nevertheless, the differences between the control and exercised neurons are preserved after isolation. (**B**) The median frequency of action potentials per minute (AP/min). Left to right: control neurons recorded in the CNS, neurons from exercised snails recorded in the CNS, measurements are performed 5 minutes after electrode penetration into the neuron (n = 32, p < 0.005), control neurons recorded after 5 min of complete isolation and neurons from exercised snails recorded after 5 min of complete isolation (n = 29, p < 0.01). Mann-Whitney test. All values are given as the median with quartiles. (**C**) Records of control (C) and exercised (E) PeA neurons after 5 min of isolation from the nervous system.

Our previous study suggested that intense muscular crawling produces an excitatory effect on the activity of serotonergic neurons of the PeA cluster controlling cilial and muscular locomotion^21^. Here, we confirmed this effect on the PeA cells in a sample of sufficient size. In the CNS preparations taken from snails which were previously forced to exercise (2 hours, Fig. 1A), PeA cluster neurons (Fig. 1B, marked with color) showed significantly enhanced firing rate compared to the control preparations (n = 32, Figs 2 and 3B, left panel). The five minute parallel records of the electric activity of the PeA8 neurons from the control and exercised snail are shown in the Fig. 2A. Below are the mean firing rate measured for 25 minutes (Fig. 2B). Similar differences can be seen also in Fig. 3A depicting the process of isolation of control and exercised neurons. Statistical analysis is provided in Fig. 3B, left panel. The differences could be observed for several hours after CNS isolation (up to 4 hours).

PeA neurons, isolated from the ganglia of exercised or control animals and kept in the physiological solution (Fig. 3A), preserved the electrical differences (n = 29, Fig. 3B, right panel). Neurons isolated from exercised snails had a depolarized membrane potential in comparison to those taken from control specimens (−50 ± 4 mV versus control −58.9 ± 4.7 mV, p < 0.01) and a higher rate of firing (Fig. 3B,C). These differences could be observed for at least one hour following their isolation.

Experimental and control neurons hyperpolarized and decreased their firing rate after their isolation (Fig. 3A,B). This observation supports previous findings suggesting strong excitatory influence of chemical microenvironment on PeA neurons in intact conditions^12,22–24^. PeA neurons have long neurites (up to 1 cm), and so during the isolation they are moved from the ganglia at least a distance of two ganglia diameters. It has been previously established that at this distance, no influence of the chemical microenvironment of ganglia is detected^20,22,23^. Therefore, isolated neurons lose both the morphological and chemical connections.

These data indicate that the experience of intense locomotion changes the biophysical properties of PeA neurons. These changes can be preserved even after the isolation of neurons from their functional network, i.e. after loss of of synaptic connections and distal parts of their neurites.

### Excitation of PeA neurons after exercise is abolished by 2 hours of rest in the CNS but not after complete cell isolation

**Figure 4:**
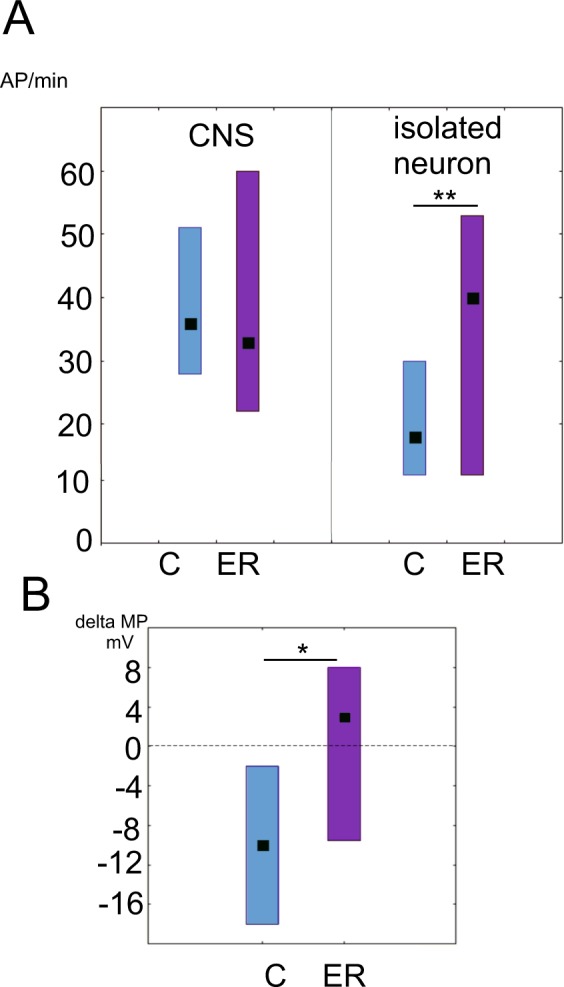
The effect of rest after exercise on the activity of PeA neurons and their response to isolation. **(A)** The median frequency of action potentials per minute (AP/min). Left to right: control neurons recorded in the CNS, ER neurons recorded in the CNS (n = 62, p = 0.5), control neurons recorded after 5 min of complete isolation and ER neurons recorded after 5 min of complete isolation (n = 43, p < 0.05). All values are given as the median with quartiles. (**B**) The median difference in the membrane potential in response to isolation in the control (left) and the ER neurons (right) (n = 43, p < 0.05). Mann-Whitney test. All values are given as the median with quartiles.

**Figure 5:**
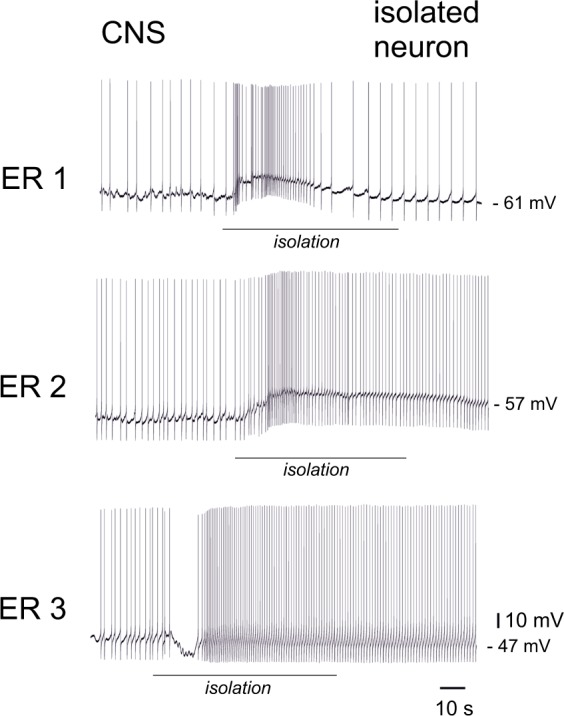
Records of electrical activity of PeA neurons during isolation from the nervous system of exercise-rested (ER) snails in three different experiments. The line indicates the mechanical isolation (electrode touch by the experimenter, pulling the neuron out of the ganglion, and moving the neuron away from the ganglion chemical microenvironment). The activity of neurons in the CNS (left of the black line), and the activity of the same neurons in complete isolation (right of the black line) is also shown. Note that the decrease in the firing rate in response to isolation, which is characteristic of control and exercised neurons (Fig. 2A) is not evident here. In the upper trace it is weaker than in the control while in the middle and low traces the opposite effect is clearly seen: excitation and an increase in the firing rate after isolation.

How long is this single cell memory maintained? In order to answer this question we first let snails “rest” for two hours after the exercise. No difference was observed in the firing rate of the PeA neurons in the ganglia dissected from exercised-rested (ER) and non-exercised control snails (Fig. 4A). However, when neurons were isolated, there was the difference in the firing rate between the control and the ER PeA cells (Fig. 4A, right panel). The neurons from the control preparations (n = 23) responded to isolation with stronger hyperpolarization compared to the neurons from ER snails (n = 20) (Figs 4B and 5). The change in MP was significantly different between the two groups: + 2.3 ± 3 mV in the ER group in contrast to −10 ± 3 mV in the control group; p = 0.003) as shown in Fig. 5B. This opposite changes in MP of ER and control neurons after isolation resulted in significant differences in the firing rate between isolated neurons from the control and exercised-rested snails (Fig. 4A).

Therefore, we encountered “hidden” differences in the endogenous electrical activity of neurons from snails with past experience of intense locomotion. What are the factors that mask these differences when neurons are recorded in the CNS remained unknown.

### Extrasynaptic release from the pedal A cluster of the control and exercised-rested snails differs

**Figure 6:**
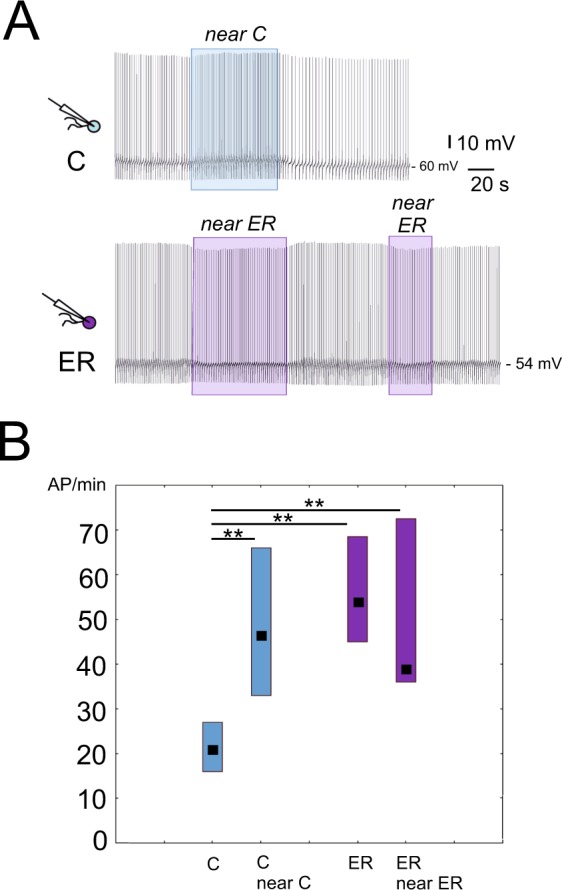
Influence of the pedal ganglia microenvironment on the activity of isolated control and ER PeA neurons. (**A**) Upper trace, record of activity of an isolated control neuron at the distance of two ganglia diameters from the PeA cluster, and near the PeA cluster of its home ganglia taken from a control snail (blue frame). Lower trace, record of activity of an isolated ER neuron in the same experiment in the same dish at the distance of two ganglia diameters from the PeA cluster, near the PeA cluster of its home ganglia taken from an ER snail (purple frames). This is an example of a clearly hyperpolarizing influence observed in several experiments. In other experiments, a depolarizing influence was observed: however, it was significantly weaker than that in the control. The vertical lines of the frames mark the end of the neuron movement. (**B**) The median frequency of action potentials per minute (AP/min). Left to right: control isolated neurons kept at a distance from ganglia, control isolated neurons near their home pedal A cluster of control snails (n = 14), ER isolated neurons at a distance from ganglia, and ER isolated neurons near their home pedal A cluster of ER snails (n = 8). All values are given as the median with quartiles. Significant differences were observed between the isolated control neuron placed at the distance of two ganglia diameters from the PeA cluster and the three other groups (Multiple comparisons test, Kruskal-Wallis test: H (3, N = 47) = 28.77 p < 0. 0015; z > 3.6 for all three comparisons). There is no difference between the other groups (z < 0.94; p > 0.1). Compare this effect with Fig. 4a illustrating the differences between a control isolated neuron and three other groups (namely, the control neurons recorded in the CNS, the ER isolated neurons and ER neurons recorded in the CNS).

Our experiments indicate that either the microenvironment or synaptic connections or both might be responsible for PeA masking in the CNS of ER snails. In the first series of experiments we tested the possible role of microenvironment: isolated neurons were moved back to their initial position in the ganglia. In accordance with the earlier data^12,22–24^, the control environment excited previously isolated control neurons (n = 14, Fig. 6). In contrast, already excited ER neurons (ER, n = 8) were not further excited by their microenvironment (Fig. 6B). In 5 out of 8 experiments, hyperpolarizing effect of ER microenvironment on ER isolated neurons was observed (Fig. 6A). A comparison of four groups, i.e., isolated control neurons in physiological solution(C), isolated control neurons near the control pedal ganglia (C near C), isolated ER neurons in physiological solution (ER), and isolated ER neurons in their home microenvironment (ER near ER), revealed a highly significant difference between group (C) and the other three groups at p level < 0.001 in all three cases. No difference between groups (ER), (ER near ER) and (C near C) was seen (Fig. 6B). Therefore, the influence of microenvironment might explain why neurons with different endogenous activity fire with a similar rate when recorded in the nervous system.

**Figure 7:**
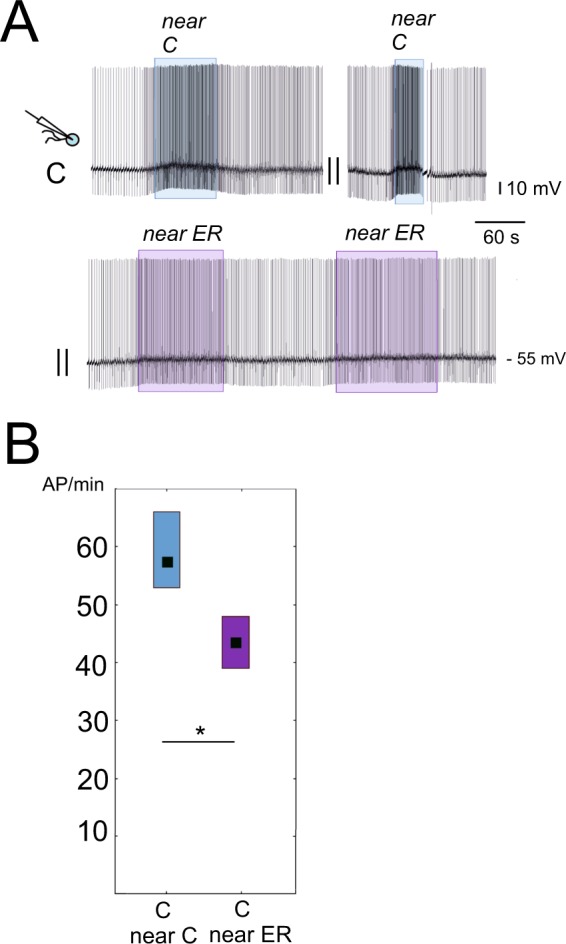
The responses of control neurons to a nearby PeA cluster of pedal ganglia taken from control and ER snails. **(A)** Upper trace, records of activity of one cell at the distance (no frames) and near the PeA cluster from the control snail (blue frames). The lower trace shows the activity of the same cell moved to the PeA cluster of ER ganglia (purple frames). The frame indicates the neuron position near the PeA cluster. It corresponds to the immobile state of the neuron. In all cases, excitation is seen when the neuron is in the PeA cluster microenvironment; however, it is significantly weaker near the ER ganglia. (**B**) The median frequency of action potentials per minute (AP/min, n = 9) near the pedal A cluster of control snails, and near the pedal A cluster of ER snails. Wilcoxon test for dependent samples shows significant differences between the responses of isolated neurons to the C and ER ganglia microenvironment (p = 0.02, z = 2.2). All values are given as the median with quartiles.

To confirm the suggested differences in the microenvironment content between ER and control ganglia, we used isolated control cells placed first near the control and then near the ER PeA cluster (the order was different in different experiments) as shown in Figs 1B and 7. In all 9 experiments with 9 neurons, the excitatory effect of the chemical microenvironment of the control pedal ganglia was stronger than that of the ER pedal ganglia. Figure 7A illustrates the responses of the same isolated neuron to the control and to the ER ganglia. An obviously weaker response to the ER pedal A cluster can be seen. There was a statistically significant difference between the effects of chemical microenvironment of control and ER pedal ganglia (Wilcoxon paired test, p = 0.02, z = 2.2, Fig. 7B). However, in no control cell an inhibitory effect of the ER microenvironment detected, in contrast to the previous series in which the firing frequency of 5 out of 8 ER isolated neurons was reduced in the ER microenvironment. The responses of the ER isolated neurons to control and ER ganglia microenvironment were tested in two experiments only. In both, they had higher rate of firing near the C ganglia.

Finally, we checked whether the weaker excitatory effect of pedal microenvironment was induced by rest rather than intense locomotion itself. The responses of the isolated control neuron placed near the PeA cluster of the control and exercised (E) snails were examined (n = 13). In this series of experiments several neurons were silent after isolation (Supplement, Fig. 1). As a result, we used the difference in the membrane potential as an indicator of the neuronal response to the ganglia microenvironment. The excitatory response was significantly stronger near the pedal ganglia of E snails (Supplement, Fig. 1). A similar tendency (not significant at p = 0.05) was observed with active cells isolated from the nervous system of exercised snails (n = 5, Supplement, Fig. 2).

Therefore, we conclude that changes in extrasynaptic release may contribute to the masking effect, described above, of rest on the firing rate of PeA neurons in CNS preparations. Although it is unlikely that extrasynaptic release completely explains the differences in the activity of neurons in intact and isolated state (either in the control, or in the experimental conditions), its impact on neuron activity in the described situation is evident.

### The dopamine receptor antagonist sulpiride unmasks the differences between the control and exercise-rested PeA neuron states in the isolated CNS

In looking for possible neurotransmitters that might explain the masking effect in the CNS of ER snails we considered the dopaminergic system. Opposite effects of serotonin and dopamine on locomotor behavior have been found in several mollusk species^25–27^. To test possible involvement of dopamine, the dopamine antagonist sulpiride (0.01–0.1 mM) was added to the dish containing CNS preparations isolated from the control and ER snails. This drug has repetitively been demonstrated to antagonize the dopamine effects in *Lymnaea*^28,19^. The PeA neurons activities were recorded simultaneously in the control and in the ER preparations prior to the drug application, during 5 minutes of sulpiride application and 20–30 minutes of washing.

**Figure 8:**
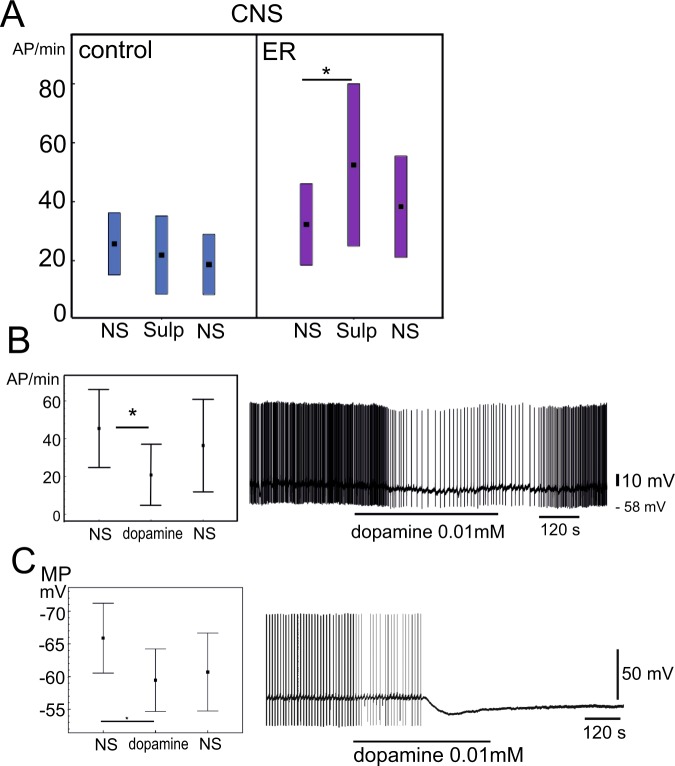
The influence of dopaminergic signaling on the activity of PeA neurons in isolated CNS from control (C) and exercised-rested (ER) snails. (**A**) The effect of dopamine receptor antagonist sulpiride (0.01 mM). Left panel: the mean frequency of action potentials per minute (AP/min) of PeA neurons from control snails (n = 9) in normal saline (NS); the activity of the same neurons after 10 min of sulpiride application (Sulp); the same after 20 min washing (NS). Right panel: the mean frequency of action potentials per minute (AP/min) of PeA neurons from exercised-rested snails (n = 9) in normal physiological saline (NS) and after 10 min of sulpiride application (Sulp); same after 20 min washing (NS). Significant differences after posthoc comparisons are marked with asterisk (ER preparations), Friedman ANOVA for multiple comparisons of dependent variables Chi Sqr = 7, p = 0 0.03). All values are given as the mean with standard deviations. (**B**) The effects of dopamine on the PeA neurons activity in the CNS. Left panel: the mean frequency of action potentials per minute (AP/min, n = 10) of PeA neurons from control snails in normal saline (NS); the activity of the same neurons after 10 min of dopamine application (dopamine); the same after 20 min washing (NS). Significant differences after posthoc comparisons are marked with asterisk, Friedman ANOVA for multiple comparisons of dependent variables Chi Sqr = 7.8, p = 0 02). Right panel, the record of PeA8 activity in the CNS of control snail prior to, during and after application of dopamine. (**C**) The effects of dopamine on the activity of isolated PeA neurons. Left panel: the membrane potential (mV, n = 10) of PeA neurons isolated from control snails in normal saline (NS); the membrane potential of the same neurons after 10 min of dopamine application (dopamine); the same after 20 min washing (NS). Significant differences are marked with asterisk, Friedman ANOVA for multiple comparisons of dependent variables Chi Sqr = 7.2, p = 0.027). Right panel, the record of isolated PeA neuron activity prior to, during and after the application of dopamine.

Sulpiride had no effect on the control PeA neurons in the CNS preparation (n = 9; Fig. 8A). Remarkably, in the CNS preparations from ER snails, it produced an excitatory effect on the PeA neurons (n = 9, Fig. 8A). These results support the data obtained in the experiments with neuron isolation suggesting increased endogenous excitation of the PeA cells in ER snails. They suggest that there is a tonic dopamine release that leads to a continuous reduction of PeA neuron activity in ER animals.

To further verify the possibility that excitatory effect of sulpiride relies on disinhibition of neurons hyperpolarized by dopamine, dopamine effects on activity of PeA neurons were tested in control preparations. Dopamine (0.01 mM) decreased the firing rate of PeA neurons (n = 10, Fig. 8B).

Whether dopamine acts on the PeA neurons directly or indirectly, or both, remained unknown. Our data indicate that it may act directly on the isolated PeA neurons (n = 10, Fig. 8C). In these experiments, dopamine at the same concentration of 0.01 mM produced hyperpolarization as well. Recent findings also suggest that the effects of exercise and rest after exercise can be reproduced in isolated paired pedal ganglia (Sultanakhmetov, Master’s Thesis, 2018). Together these data suggest that dopamine might indeed be responsible for the changes observed in the microenvironment of these ganglia in ER snails.

We conclude that an increased tone of the dopaminergic system in the CNS of ER snails is likely to be responsible for masking the excitatory state of the serotonergic PeA neurons. The precise mechanisms of its action as well as the possible cellular sources of an increased dopamine tone during ER state need further investigation.

### ER snails show faster locomotor arousal on dry surface

The ambiguous state, characterized by both keeping memory of the past and adjusting to the present context, is interesting in its potential to return rapidly to a previous state of enhanced activity, if necessary. We tested whether locomotor behavior of the ER and control snails (n = 30; 30) differs when animals are taken from water and placed on a dry surface. We used the same procedure as in the earlier paper^21^. Snails were placed in the asymmetrically lit dry arena. Two minutes later, their speed of locomotion was analyzed for four minutes with the Ethovision program. ER snails showed faster locomotion than the control ones with the median speed 2.5 cm/min versus 1.9 cm/min in the control group (z = 2.18; p < 0.03; Mann-Whitney U Test). This finding is in line with the suggestion that the endogenous excitation of PeA neurons in the ER snails may underlay a faster behavioral switch from aquatic to terrestrial crawling.

## Discussion

Predictions from the past about the future are important for survival^29,30^. However, relying on the past may turn out to be erroneous in certain circumstances. How should one know when it is time to stop relying on the past and to make models of the future relying on the present? How should one make a decision when the past and present experiences contradict? This task is very difficult and very important for all living organisms, with no exception. It is the cause with some psychological and even some psychiatric problems in humans.

In very simple and common cases, the longer the circumstances related to the past experience are absent, the more likely an organism will exclude them from its “predictive model of the external world”. Here, we addressed the questions of how past and more recent experiences interact on the level of a single neuron. In the gastropod snail *Lymnaea stagnalis*, which is useful for studies of freshly isolated neurons, we found that a single isolated neuron is capable of storing the memory about its activity during the past behavioral state. Second, we discovered that this persistent memory can be masked by the nervous system when newer information becomes available. We propose that the neuroactive chemical microenvironment and specifically, an increased content of dopamine, plays a role in the adjustment of serotonergic neurons that were modified by previous experience to novel circumstances.

The PeA cluster serotonergic neurons are involved in the modulatory control of locomotion. The cells deliver serotonin to the ciliated epithelium and foot muscles of *Lymnaea*^31,32^. Excitation of pedal serotonin neurons is associated with the locomotor arousal (swimming) in the marine gastropod mollusks *Aplysia fasciata* and *Clione limacina*^33–35^. In several distantly-related mollusk species, the serotonergic neurons have excitatory chemical and electrical interconnections. It was suggested that they form a “distributed arousal network” that may underlie the locomotor arousal of the animal^36–39^. Since the extrasynaptic release of serotonin from the PeA neurons within the central ganglia of *Lymnaea* was found, a wider neuromodulatory role was ascribed to these cells^12,20,22,23^. Serotonin is known to facilitate many forms of behavior beyond locomotion, including cognitive traits such as learning and memory in mollusks^18,39,40^.

Here, we found that previous motor load is represented in the electrical properties of isolated serotonergic neurons. Earlier, we demonstrated a similar representation of hunger in the activity of isolated PeA neurons^12^. Locomotor arousal is typically observed in hungry animals, including mollusks^41^. It results in random or directional food-seeking behavior. The increased excitatory state of “hungry” and “exercised” PeA neurons corresponds to their functional role in natural behavior. Together, these findings clearly show that memory of recent activity is stored in the neurons and, at least in some stages, does not require network involvement.

A cellular “set of memory” has been broadly discussed during recent years. The common point of view that memory is represented in synaptic strength has been criticized recently by several authors. Memory was suggested to be encoded on the inside of neurons^7–18,42–44^^,^ in the cellular microenvironment^45,46^, and in unique neuronal ensembles^47^. In mollusks, a persistent depolarization of membrane potential was demonstrated to contribute to a long-term associative memory trace^16–18^.

Our results, on the one hand, provide the strongest support to the idea that the memory of previous activity can be stored inside of the neuron. On the other hand, we unveil the complex interactions between a single neuronal memory and a system “knowledge” of the current situation. We demonstrated that memory of past activity is preserved within a neuron and does not require ensemble effects at a certain stage. In comparison to previously reported forms of memory revealed in delicate changes of synaptic strength, our “excited after isolation” neuron is probably one of the boldest and simple examples of how previous experience can be stored.

The masking effect of the nervous system on the increased activity of ER neurons is probably the most interesting finding presented in this work. The difference in the firing rate between the control and the ER PeA neurons not seen in the ganglia became apparent when the neurons were isolated. The membrane potential of ER neurons hyperpolarized more weakly than the membrane potential of control neurons after isolation, which is consistent with and partially explains this effect. These findings suggest that not only do the inner properties of pedal neurons differ between the control and ER snails, but so does the impact of the neuron network on these cells. This impact seems to be able to compensate for the differences between the control and ER neurons. It is likely that dopamine may play a key role in this masking effect, since its antagonist produced excitation in the PeA neurons from ER snails, and had no effect in the control group. In other words, we encountered a peculiar situation characterized by the seemingly equal activity of neurons in the CNS, which was maintained by different mechanisms.

This finding imposes an obvious question: what is the physiological reason for keeping locomotor neurons in an internally excited state under external inhibition in ER animals? We suggested that this ambiguous state, characterized by both keeping memory of the past and adjusting to the present context, is interesting in its potential to return rapidly to a previous state of enhanced activity, if necessary. Indeed, ER snails showed faster locomotor arousal when they were placed again in terrestrial conditions. This finding points to possible benefits of this ambiguous state for transition from aquatic to terrestrial locomotion. It also agrees with presumption that the memory of the past is still used for predictive models of a possible future.

There is a growing understanding that the neuromodulatory microenvironment of a network is not less important than the connections in the functional physiology of the nervous system. The role of neuromodulation in neural mechanisms underlying decision-making has been demonstrated in many studies^48,49^. It is well established that in addition to synaptic interactions, there is a broad range of nonsynaptic chemical communication between neurons. Extrasynaptic neurotransmitter release is proven to play an important role in the nervous system of mammals^50–55^ and various invertebrates^19,20,22–24,56,57^. Recently, changes in the extrasynaptic modulatory state were shown to be associated with different behavioral states^12^. Still, we know surprisingly little about the contribution of nonsynaptic communication to memory formation.

Here, for the first time, we found evidence that changes in the extrasynaptic release can contribute to a peculiar masking effect of the network on the persistent memory of past behavioral experience in individual neurons. We found that the difference in the firing rate observed between control and ER isolated neurons was masked when these neurons were placed in their home microenvironment. This effect is remarkably consistent with the absence of difference between these neurons when the measurement are made while these are in the ganglia. The control isolated neurons with lower endogenous activity responded to their home ganglia microenvironment with profound excitation, while the ER isolated neurons with a higher firing rate either demonstrated the opposite inhibitory response to their ganglia microenvironment or a significantly weaker excitatory one.

The difference in the extrasynaptic release between the control and ER ganglia was confirmed when the same cells were used to detect the activity of the microenvironment of both preparations. They similarly demonstrated significantly weaker excitation near the ER ganglia. It can be noticed, however, that in this experimental series we never observed the inhibitory effect of the ER ganglia. This may potentially indicate that not only the microenvironment but also the receptiveness of the neurons from the ER snails was changed. It was not the aim of the present study to establish this, but it can be an interesting task for further investigation.

Finally, we checked whether the shift in the balance between the excitatory-inhibitory components in the ER microenvironment was induced by the return to aquatic conditions. We compared the responses of isolated neurons near the control and the exercised (E) ganglia. The effect on the E and ER ganglia microenvironment was completely different. The neurons detected an even stronger excitatory influence of the E ganglia compared to that of the control ones. This result agrees with the idea that an increase in the inhibitory influence of the microenvironment is induced by a cessation of intense locomotion and a return to aquatic conditions.

**Figure 9:**
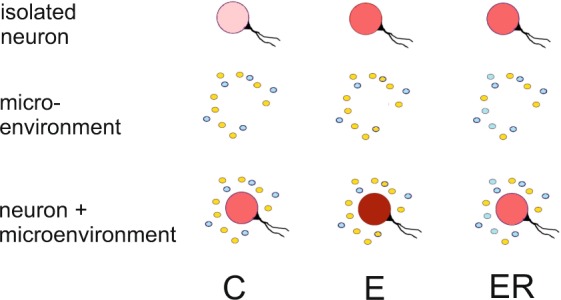
Schematic representation of two mechanisms in the interplay of past and present experiences at the cellular level: (1, upper panel) intrinsic neuronal changes in the biophysical properties of the cell membrane and (2, medium panel) extrinsic neuronal changes in the extrasynaptic microenvironment of the ganglia. Exercise (E) results in an enhanced firing rate (marked with red color of different intensity) of individual neurons and a stronger excitatory influence of the microenvironment (marked with yellow color), while rest following the exercise (ER) enhances the inhibitory extrasynaptic influence (marked with blue color) on still-excited individual neurons. The latter results in nearly equal activity of the control and the ER neurons in the CNS (lower panel). However, this similarity is explained by totally different states of both the neurons and their chemical environment. Different intensity of red color reflects the difference in the rate of firing of PeA neurons, with more intense color corresponding to higher activity. Balance between excitatory (yellow) and inhibitory (blue) neurotransmitters/neuromodulators is suggested to define the polarizing effect of microenvironment.

In conclusion, we show the involvement of the two mechanisms in the interplay of past and present experiences at the cellular level (Fig. 9): (1) intrinsic neuronal changes in the biophysical properties of the cell membrane and (2) extrinsic neuronal changes in the extrasynaptic microenvironment of the pedal ganglia. Exercise results in an enhanced firing rate of individual neurons and a stronger excitatory influence of the microenvironment, while rest following exercise enhances the inhibitory extrasynaptic influence (presumably via dopamine release) on still-excited individual neurons. The latter results in nearly equal activity of the control and the ER neurons in the CNS. However, this similarity is explained by totally different states of both the neurons and their chemical environment. The results agree with the idea proposed for central pattern generators and supported by mathematical modeling that “multiple solutions produce similar outputs”^58,59^. We hypothesize that the same output (behavior) can be produced by circuits with different combinations of neuron parameters depending upon the past experience of the animal and its expectations about the future. ‘The right combination’ may facilitate the transition from the current behavior to the predicted one.

## Materials and Methods

### Animals

Mature snails *Lymnaea stagnalis* were obtained from a breeding colony. The colony originated from mixed groups of wild animals collected in the Oka river, Moscow region, in 1992–1998. Animals were kept in dechlorinated tap water at room temperature and fed on lettuce *ad libitum*.

Enhanced motor activity was evoked as in^21^ by putting snails for two hours into a 25 × 50 cm tank filled with 1 mm of water which prevented the mollusks from drying but stimulated them to perform intense terrestrial-like muscular locomotion (Fig. 1A). Control snails were kept in deep water so they could use ciliary locomotion for two hours in similar light conditions. “Rest after exercise” was evoked by putting snails for two hours after motor activity into a cylinder filled with water. The experimental and control animals were chosen at random and tested in one experiment at the same time.

### Electrophysiological experiments and neuron isolation

Standard procedure described previously in^12,19^ was used. In each experiment, the central ganglia were dissected from two animals (control and experimental), anesthetized with an injection of 0.1 mM MgCl_2_. The central ganglia (with exception of buccal ones) were placed into a 2.5 mg/ml solution of pronase E (Sigma) for 15 minutes, washed in a standard snail Ringer’s solution (50 mM NaCl, 1.6 mM KCl, 4 mM CaCl_2_, 8 mM MgCl_2_, 10 mM Tris, pH 7.6), and pinned to a Sylgard in a four-milliliter chamber with a distance of approximately 1 cm between preparations. The connective tissue sheath was then removed from the pedal ganglia.

Visual identification of the PeA2/A8 neurons was performed based on their location, size and color. Other neurons were randomly taken from serotonergic Pedal A (PeA) clusters (Fig. 1B, marked with color) to assess whether the observed effects of motor load were common to different cluster members. In terrestrial snails the serotonergic neurons of the pedal cluster were shown to produce different secreted and non-secreted peptides^60^.Whether Lymnaea PeA cells co-express different peptides is unknown.

The neuron that was selected for examination was impaledpenetrated with a standard glass microelectrode (10–20 M filled with three molar KCl). A standard setup for microelectrode recording was used. The electrophysiological recordings were stored in computer files using a home-developed program.

For neuron isolation, we utilized previously developed methods^61,62^. The neuron was gently pulled out of the tissue using the intracellular microelectrode until separation of the proximal neurite from the neuropil was achieved. The electrical activity of the cell was monitored during isolation. The cells that demonstrated membrane injury were not used for the experiments.

### Investigation of modulatory effects of the pedal ganglia microenvironment on the electric properties of PeA neurons

Our approach was developed based on the earlier methods for the detection of extrasynaptic release from the ganglia of *Lymnaea*^12^. Preparations of central ganglia were used in one experiment and placed in the same chamber with a distance of approximately 1 cm between them. One nervous system was used as a source of isolated neurons and was treated as above (the neuron isolation procedure). The positions of control and experimental preparations in the chamber were altered in different experiments, and the investigator was not aware of where the control and experimental preparations were placed (“blind procedure”). The connective tissue sheath was removed from the pedal ganglia.

The isolated neuron penetrated with the microelectrode was moved away from the pedal ganglion and placed in the middle between the control and experimental pedal ganglia for two minutes (Fig. 1B). After that two approaches were used. (1) Neurons isolated from the control and experimental preparations were moved back close to their positions in their home ganglia. (2) Isolated neuron was first moved to the pedal cluster of experimental preparation at a distance less than half-cell size (20–25 µm) and kept in this position for up to two minutes, placed at the distance from the ganglia, then moved to the PeA cluster of the control preparation of ganglia. The procedure was repeated several times.

### Data analysis

The significance of the differences was subjected to the Mann-Whitney test (the differences in spike frequency and membrane potential between control and experimental neurons *in situ* and in isolation) or by the paired Wilcoxon signed-rank test for dependent samples (the differences in the activity of neurons near the control and experimental ganglia) or the multiple comparisons test (Kruskal-ANOVA for independent and Friedman ANOVA for dependent variables) for multiple comparisons with posthoc tests using the STATISTICA program (StatSoft Inc.). All values are given as medians with the upper and lower quartiles.

### Highlights

We addressed the question of how past and present behavioral experience interacts at the level of a single neuron. Using the pond snail *Lymnaea stagnalis*, we found that a single isolated neuron is capable of storing the memory about its activity during the past behavioral state. However, this persistent memory of an individual neuron can be masked by the nervous system when newer information becomes available. We show that the chemical microenvironment plays a role in the adjustment of neurons that were modified by previous experience to novel circumstances.

## Supplementary information

Dataset 1

## Data Availability

The datasets generated and/or analyzed during the current study are available from the corresponding author on reasonable request.
