# Supplementary material for: Storage and erasure of behavioural experiences at the single neuron level
Source: Sci Rep. 2019 Oct 14;9:14733. doi: 10.1038/s41598-019-51331-5 (PMC6791831; doi:10.1038/s41598-019-51331-5)

Storage and erasure of behavioural experiences at the single neuron level

T.L Dyakonova, G. S. Sultanakhmetov, M.I. Mezheritskiy, D. A. Sakharov, V.E. Dyakonova*

Figure1. The responses of biosensors to nearby PeA clusters of pedal ganglia taken from control and exercised (E) snails. **(a)** Record of the membrane potential of a biosensor (isolated control PeA neuron) at the distant point and near the PeA cluster from an E snail (red frames, left and right), and near the PeA cluster from a control snail (middle, blue frame). (**b**) The median difference in the membrane potential level of biosensors in response to proximity to the control (left) and the E (right) PeA clusters. ($n=13$, $p<0.05$). Wilcoxon test for dependent samples. All values are given as the median with quartiles.

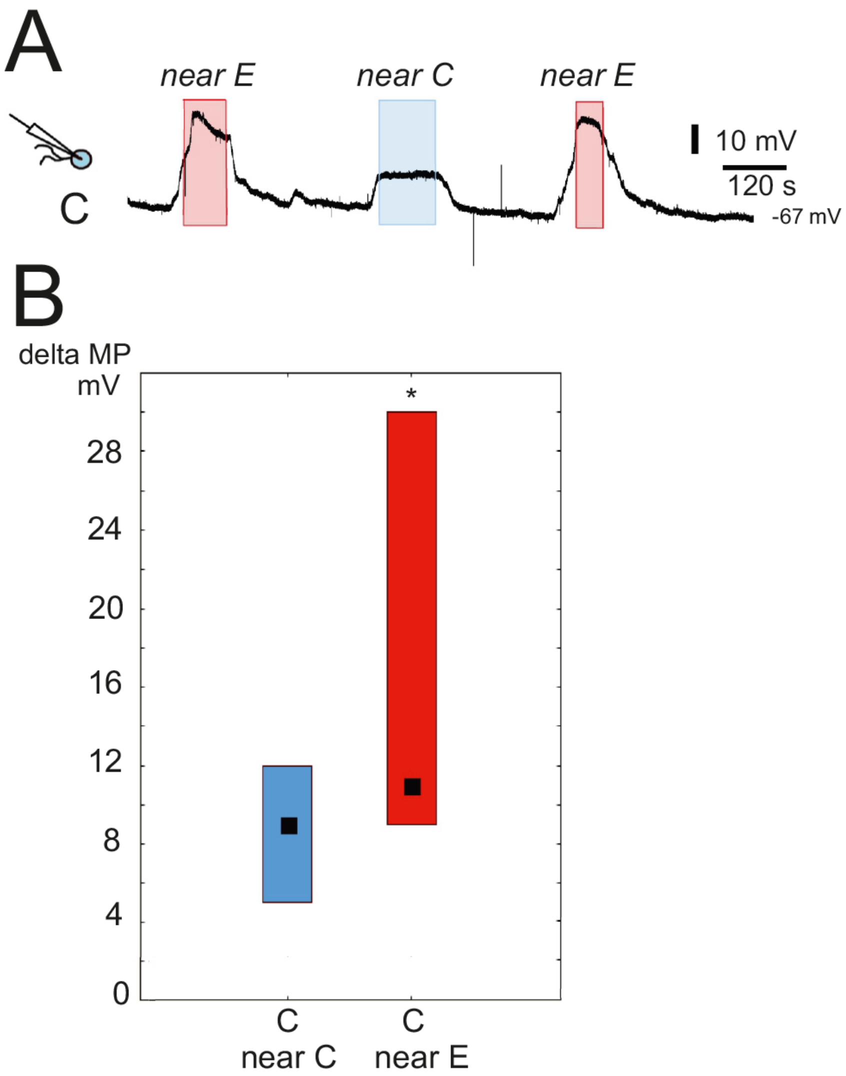

Figure 2. The responses of neurons isolated from exercised snails to nearby PeA clusters of pedal ganglia taken from control (C) and exercised (E) snails. **(a)** Record of activity of isolated E neuron at the zero point and near the PeA cluster from an exercised (E) snail (red frames, left and right), and near the PeA cluster from a control (C) snail (middle, blue frame). (**b**) The median frequency of action potentials per minute (AP/min, n=5) near the pedal A cluster of control snails, and near the pedal A cluster of E snails. All values are given as the median with quartiles.

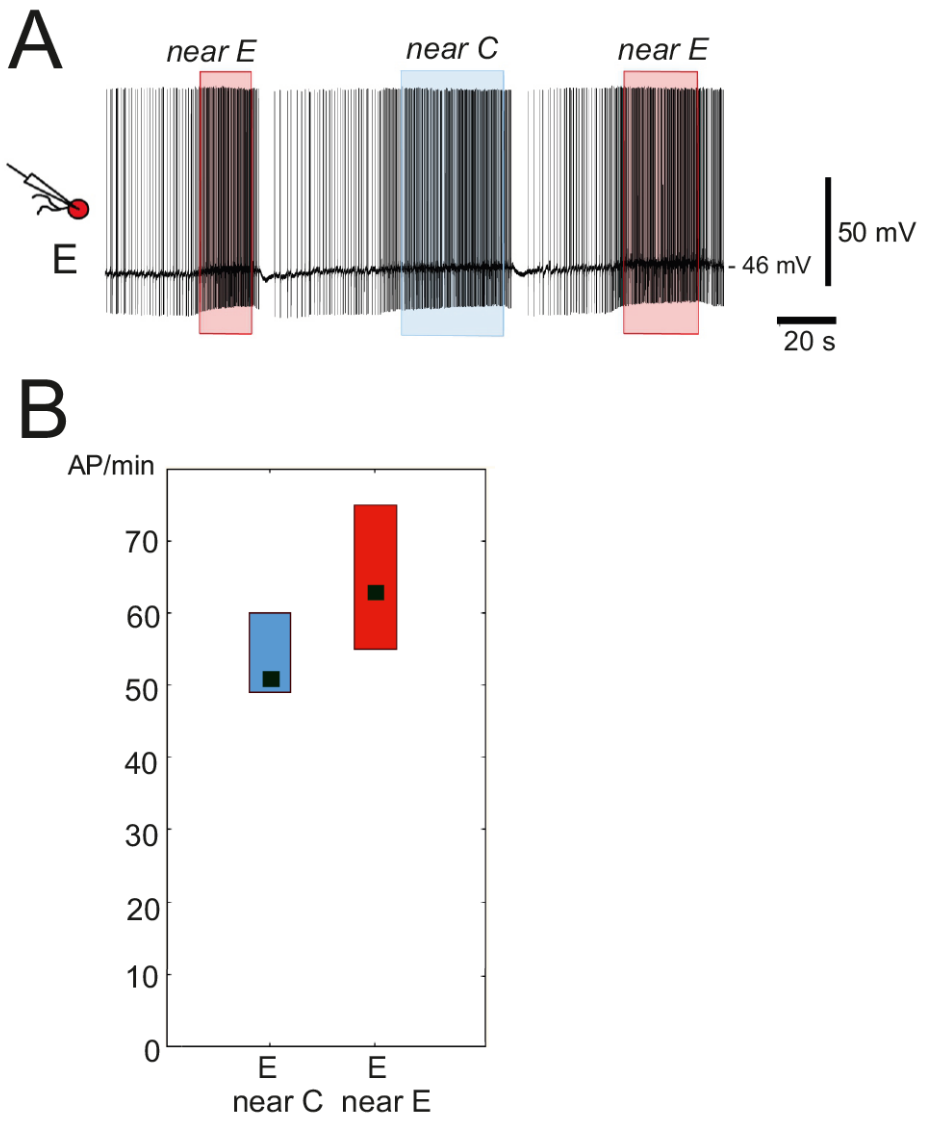

Supplement: Supplementary file 1 — Dataset 1 [file 41598_2019_51331_MOESM1_ESM.pdf]
